# Application of Radio Environment Map Reconstruction Techniques to Platoon-based Cellular V2X Communications

**DOI:** 10.3390/s20092440

**Published:** 2020-04-25

**Authors:** Sandra Roger, Carmen Botella, Juan J. Pérez-Solano, Joaquin Perez

**Affiliations:** 1 Computer Science Department, Universitat de València, Av. de la Universitat s/n, 46100 Burjassot, Spain; carmen.botella@uv.es (C.B.); juan.j.perez@uv.es (J.J.P.-S.); 2 Department of Electronic Engineering, Universitat de València, Av. de la Universitat s/n, 46100 Burjassot, Spain; joaquin.perez-soler@uv.es

**Keywords:** vehicular communications, intelligent transportation systems, radio environment maps, channel acquisition, spatial interpolation, Kriging, 5G

## Abstract

Vehicle platoons involve groups of vehicles travelling together at a constant inter-vehicle distance, with different common benefits such as increasing road efficiency and fuel saving. Vehicle platooning requires highly reliable wireless communications to keep the group structure and carry out coordinated maneuvers in a safe manner. Focusing on infrastructure-assisted cellular vehicle to anything (V2X) communications, the amount of control information to be exchanged between each platoon vehicle and the base station is a critical factor affecting the communication latency. This paper exploits the particular structure and characteristics of platooning to decrease the control information exchange necessary for the channel acquisition stage. More precisely, a scheme based on radio environment map (REM) reconstruction is proposed, where geo-localized received power values are available at only a subset of platoon vehicles, while large-scale channel parameters estimates for the rest of platoon members are provided through the application of spatial Ordinary Kriging (OK) interpolation. Distinctive features of the vehicle platooning use case are explored, such as the optimal patterns of vehicles within the platoon with available REM values for improving the quality of the reconstruction, the need for an accurate semivariogram modeling in OK, or the communication cost when establishing a centralized or a distributed architecture for achieving REM reconstruction. The evaluation results show that OK is able to reconstruct the REM in the platoon with acceptable mean squared estimation error, while reducing the control information for REM acquisition in up to 64% in the best-case scenario.

## 1. Introduction

In upcoming years, the transport industry is expected to witness an intense period of change and evolution thanks to the implementation of intelligent transportation systems (ITSs). These systems rely on wireless communications and new computing and sensor technologies to provide a more efficient, safe and comfortable mobility in a variety of vehicular scenarios. In particular, vehicle to vehicle (V2V) and vehicle to infrastructure communications (V2I), generally encompassed under the term vehicle to anything communication (V2X), are fundamental for different purposes such as the prevention of collisions between vehicles, emergency notification to distant vehicles, route and track optimization, and automated driving. In this direction, the Third Generation Partnership Project (3GPP) defined the term “enhanced support of V2X communication services”, known as eV2X [1], which targets several use cases such as advanced driving (semi- or fully automated driving), vehicle platooning [2], extended sensors, and remote driving. In addition to the need for very high reliability, these use cases require low latency communication (in the order of tens of milliseconds) [3], which can not be generally guaranteed by the main existing wireless technologies. For instance, the LTE-Advanced standard was not specifically designed for latency-critical applications such as V2X, being the required latency and reliability at system-level only fulfilled by direct communication between vehicles [4]. As a consequence, the drawbacks of current options for V2X communication have led the transport industry to wait for the complete definition and roll-out of 5G [5] and beyond 5G technologies, before implementing the more advanced use cases involving, for instance, autonomous vehicles.

Some autonomous-driving related services require communication among groups of users. For instance, in vehicle platooning, there are two types of platoon nodes known as head/leader (leading vehicle), and follower (the rest of the vehicles). Once the platoon is established, each vehicle must keep a reliable communication within the platoon in order to keep the group structure and carry out coordinated maneuvers [6]. Vehicular communications in groups are also fundamental for cooperative collision avoidance [3] or cooperative lane merging among nearby vehicles. The existence of redundant or correlated information in groups of vehicles (e.g., related to channel busy ratio or beam information) was exploited in [7] to propose several schemes intended to reduce the amount of signaling exchanged between vehicles and the base station (BS), thus reducing the transmission overhead in the V2X cellular communication system.

In this paper, we elaborate on the benefits of exploiting the particular features of platooning in order to decrease the control information exchange required in the V2X channel acquisition stage, with the focus of reducing the latency while ensuring the reliability of the communication. The possibility of achieving reliable channel state information at each vehicle at a reduced cost would pave the way towards enabling advanced physical-layer functionalities for the entire platoon such as the mobile relay paradigm [8]. In this particular use case, the entire platoon could act as a relay, cooperating to increase the quality of the communication with other vehicles located in poor coverage areas. A second use case would comprise enhanced intra-platoon communications, where some vehicles could act as intra-platoon relays to boost the quality of the communication for other platoon vehicles through device-to-device communications [9]. To this end, a scheme based on radio environment map (REM) reconstruction is proposed in this work, where the actual REM values are available at a subset of the vehicles, providing estimates of the large-scale channel parameters for the rest of vehicles in the platoon. In this regard, spatial channel interpolation approaches have recently gained interest in 5G automotive applications due to their ability to reconstruct the REM of a given BS from a subset of available measurements [10]. Although REMs were originally proposed as a solution for cognitive radio systems [11], they are currently considered as a real enabler for radio environmental awareness [12]. REMs are seen in the spectrum sharing scenario as databases containing the information of primary users (transmission power, location, spectrum sharing criteria). Hence, by querying the databases, secondary users can access essential information [12]. Applications extending the use of REMs cover scenarios seminal to 5G such as interference management [13], coverage analysis [14], and proactive resource allocation in anticipatory networks [15], among others. In these cases, REMs are assumed to contain information about the radio signal strength, delay spread or interference levels besides geo-localized information (position of the measurements). In the platooning use case, REMs can be included in what is known as context-aware databases, for example, to achieve a dynamic and reliable intra-platoon communication as in [16].

Traditionally, one of the key tasks in REM reconstruction is deciding an spatial interpolation method offering a good quality and complexity trade-off when the number of locations (or samples) to be considered increases. The most popular techniques in the literature are nearest neighbor [17], inverse distance weighting [17,18], natural neighbor [19], thin plate splines [19], Gaussian process regression [20,21] and Kriging [10,14,17,18,19,22,23,24]. Kriging is a method that was originally used in geostatistics, but it has been applied since then in many fields, and it is actually one of the most frequently applied methods for REM reconstruction. Literature on spatial interpolation methods has previously proved the superior performance of Kriging versus nearest neighbor and inverse distance weighting techniques under different evaluations metrics (see [17,22]) or with respect to natural neighbor (see [10]). Note that, in general, it is not straightforward to synthesize a common ground for the aforementioned interpolation schemes since different assumptions and scenarios are assumed. However, inverse distance weighting and Kriging are the most popular choices, being the former deterministic by nature and more robust, and statistical the latter. Reference [25] extended the features of inverse distance weighting to include some statistical knowledge in order to overcome the limitations of this method. In their analysis, the authors of [25] found that inverse distance weighting is sensitive to the number of samples and the exponent factor used in the model, so a careful design was needed given a certain scenario. Given this, and considering the superior results obtained by Kriging versus inverse distance weighting in [17,22] both for simulated and real-world measurements, respectively, the statistical method Kriging is the one employed in this paper.

The first step in Kriging interpolation is to form an empirical semivariogram, which is later fitted to obtain a theoretical model. The semivariogram model basically characterizes the spatial correlation of the field. In a second step, interpolation is applied by using a weighted average of known field samples to estimate the value of the field in any position. The weights are obtained by minimizing the Kriging variance (best linear unbiased estimation) and hence, the better the semivariogram modeling, the better the Kriging interpolation. Reference [19] points out the need of a central node for fusion, since Kriging is a global interpolation method, while methods such as the natural neighbor can be regarded as distributed. However, distributed implementations of Kriging can be found for example in [23,26], while reference [14] proposed fixed rank Kriging with the aim of lowering the complexity of the spatial interpolation. This paper explores the capability of Ordinary Kriging (OK) as a spatial interpolation method in the V2X platoon use case. Note that advanced versions of Kriging such as Regression Kriging could also be applied in this framework [10].

In our target scenario, V2X platoon communications, the REM of interest is reduced to a subset of positions matching the platoon area, and it comprises samples of geo-localized received power due to large-scale channel effects, i.e., path-loss and shadowing. In this framework, we evaluate the potential of OK to reconstruct the REM within the platoon when only a given number of actual REM values are available. The aim is to assess the quality of the reconstructed REM values with different degrees of signaling reduction.

The main contributions of this paper are:

- a scheme to reduce the control information exchanged for REM reconstruction in platooning based on OK is proposed. The REM reconstruction capability is evaluated via the mean square error and traded-off versus signaling overhead;
- a suitable semivariogram modeling for OK interpolation in the scenarios under consideration is proposed;
- an analysis of the optimal patterns of vehicles’ positions for reliable REM reconstruction is performed;
- expressions for the communication cost related to the REM reconstruction are derived for a centralized and a distributed architecture.

The remainder of the paper is organized as follows. Section 2 introduces the system model considered in this work. Section 3 discusses the semivariogram modeling. Simulation results are presented in Section 4, and conclusions and future work are drawn in Section 5.

## 2. System Model

In this section, the use case and scenario are first presented, corresponding to two particular vehicular platoon setups referred to as the asymmetric and symmetric scenarios. Second, the theoretical framework of OK is briefly reviewed, and the REM modeling is stated (path-loss and shadowing models). Finally, two possible architectures towards the REM reconstruction, namely, centralized and distributed, are analyzed in terms of communication cost.

### 2.1. Use Case and Scenario

**Figure 1:**
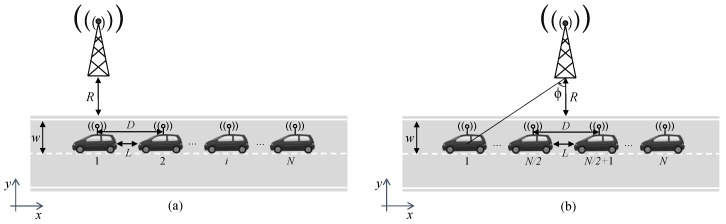
Scenarios under consideration comprising a platoon of vehicles assisted by a base station (BS). (**a**) Asymmetric case, where the BS is aligned with the antenna of the platoon leader. (**b**) Symmetric case, where the line connecting the BS with the platoon leader forms an angle ϕ with the perpendicular line, so that the BS is aligned with the platoon middle point.

The particular use case of a *N*-vehicle platoon is targeted, with each vehicle of length *l*, passing through a BS assisting the platooning communication service. Figure 1 depicts the two scenarios addressed in this paper, representing two particular platoon positions along the direction of the road (x-axis). These two scenarios are analyzed separately due to their impact on the semivariogram modeling step, which will be later discussed in Section 3. In fact, the asymmetric scenario is considered as a representative scenario for the rest of many possible platoon positions where the semivariogram modeling fulfils an increasing trend.

Figure 1a presents a situation where the BS is aligned with the antenna of the platoon leader. Since, in this case, each vehicle is located at a different distance from the BS, it is referred to as the asymmetric case. In Figure 1b, the line connecting the BS with the platoon leader forms an angle ϕ with the perpendicular line, so that the BS is aligned with the platoon middle point in the x-axis. This scenario is referred to as symmetric since half of the vehicles are located at the same distance from the BS than the other half.

Both in the asymmetric and symmetric case, the BS is located at the origin of coordinates and at a distance *R* from the road border. All platoon vehicles are assumed to be perfectly aligned, driving on the centre of the lane and separated of each other a distance equal to *L* (platoon inter-vehicle distance). The vehicle antennas are located in the middle of the roof, assuming, for simplicity, that their position matches the middle point of the vehicle in both directions. As a result, the antennas of two consecutive vehicles are separated a distance equal to D=L+l. Finally, lane width is denoted by *w*. This scenario is usually considered as a benchmark for highway communication in the V2X framework [27]. Note that, throughout the paper, we will refer to vehicle positions or antenna positions indistinctly, that is, when referring to the vehicle position, the antenna position is actually considered. With the assumptions above, the angle between the BS and vehicle #i can be calculated as:

$$\phi_{i}=\arctan\left(\frac{D(i-1)}{R+w/2}\right),\;i=1,2,\ldots ,N.$$

Since in the asymmetric scenario the BS is directly aligned with the platoon leader, the angle is $\phi =\phi_{1}=0$. In the symmetric scenario, it can be shown that the angle ϕ depends on *N* as:

$$\phi =\arctan\left(\frac{D(N-1)/2}{R+w/2}\right),$$

which holds for both odd and even *N* values. Note that this analysis could be extended by using stochastic geometry and assigning a given probability density function to the relative angle ϕ.

### 2.2. REM Reconstruction with OK

Let us assume that a minimum number of *P* field values are available at certain vehicles of the reference scenarios depicted in Figure 1. Available field values, i.e., received power due to large-scale channel parameters in this work, can be acquired either through a conventional channel acquisition stage, or through queries to a previously stored database containing the REM of the area. The aim is to perform OK to achieve REM reconstruction for the rest of the platoon vehicles (N−P vehicle locations). Both a centralized and a distributed architecture are explored in this contribution, as further elaborated in the next subsection. Interestingly, although distributed architectures generally require an initial cluster formation stage [26], the vehicular service group (platoon) can be naturally adopted as an estimation cluster. The field value available at a vehicle located at position $x_{i}$ is denoted by $V\left(x_{i}\right),\;i=\left\{1,2,\ldots ,N\right\}$. The set S includes the $x_{0}$ positions where the REM needs to be reconstructed, and its cardinality is given by |S|=N−P. These reconstructed field values are denoted by $\hat{V}\left(x_{0}\right)$.

Two steps are required in OK interpolation: semivariogram analysis and Kriging prediction. First, an empirical semivariogram (EV) is formed, which is defined as one half the average squared difference between the field values at points separated by a lag distance [28]:

(1) $$\hat{\gamma }\left(h\right)=\frac{1}{2|N(h)|}\sum_{N(h)}{\left(V\left(x_{i}\right)-V\left(x_{j}\right)\right)}^{2},$$

where $h=x_{i}-x_{j}$ is the lag distance, and $V(x_{i})$ and $V(x_{j})$ are field values at spatial locations $x_{i}$ and $x_{j}$, respectively. N(h) is the set comprising all location pairs $\left(x_{i},x_{j}\right)$ such that $x_{i}-x_{j}=h$, and |N(h)| denotes its cardinality. The EV models the correlation between field values and it is fitted to obtain a theoretical parametric semivariogram model, which is an essential step in our scenario. The semivariogram model is a mathematical expression that characterizes the trend in the EV. In a second step, OK prediction is performed, where the reconstructed field value at target location $x_{0}$ is given by:

(2) $$\hat{V}\left(x_{0}\right){|}_{P}=\sum_{i=1}^{P}w_{i|P}\left(x_{0}\right)V\left(x_{i}\right),$$

where *P* is the number of available field values, $w_{i|P}\left(x_{0}\right)$ is the weight assigned to the vehicle located at position $x_{i}$ when interpolating using *P* field values, and $\hat{V}\left(x_{0}\right){|}_{P}$ is the reconstructed field value. Equation (2) can be solved by rewriting the equations into matrix form, as derived in [10]. Vehicles are assumed to be equipped with GPS, simplifying the lag distances calculation required for the EV from Equation (1).

In this paper, the field value corresponds to large-scale channel parameters, and it is given by:

(3) $$V\left(x_{i}\right)=P\left(x_{i}\right)+S\left(x_{i}\right),$$

where $P(x_{i})$ is the average received power depending on the path-loss model and $S(x_{i})$ is the shadow fading following a zero mean lognormal distribution [29]. In the model we assume that the shadow fading is spatially correlated, with the correlation coefficient between the shadow fading at locations $x_{i}$ and $x_{j}$ defined by [30]:

(4) $$\rho_{i,j}=\;E\left[S\left(x_{i}\right)S\left(x_{j}\right)\right]=\sigma^{2}\exp\left(-\frac{∥x_{i}-x_{j}∥}{L_{corr}}\right),$$

where $L_{corr}$ is the correlation distance in meters, which is defined as the distance satisfying $\rho_{i,j}=0.5$ and $\sigma^{2}$ is the variance of the shadow fading. The average received power (dBm) at location $x_{i}$ from a single antenna BS is calculated by the simplified path-loss model:

(5) $$P\left(x_{i}\right)=P_{t}+K_{dB}+10\alpha \log_{10}\left(\frac{d_{0}}{d_{i}}\right),$$

where $P_{t}$ is the transmitted power (dBm), $K_{dB}$ is the constant path-gain factor in dB units at a reference distance $d_{0}$, α is the path-loss exponent, and $d_{i}$ is the distance between the vehicle location $x_{i}$ and the BS location. Note that, in this contribution, the small-scale fading effect is assumed to be averaged out by the receiver (see [31] for a discussion about this aspect). Hence, from now on, REM reconstruction or channel estimation in this paper will refer to the acquisition or estimation of the large-scale channel effects (only path-loss and shadowing values).

### 2.3. Centralized vs. Distributed Architecture

Two different architectures can be contemplated for the purpose of REM reconstruction via OK interpolation, namely, centralized and distributed. Note that this differentiation is aligned with the need to reduce Kriging’s complexity for large sets of samples. In the centralized setting, vehicles with available REM values forward them to the BS for reconstructing through Equation (2) in a centralized manner. In the distributed architecture, the reconstruction in Equation (2) is directly calculated at the remaining N−P vehicles by message passing among the vehicles in the group.

Assuming that both architectures lead to the same channel estimates, i.e., disregarding errors in the signaling stage, their main difference relies on the message exchange procedure, with a direct impact on the communication cost [32,33,34]. Models for the communication cost in a similar use case were recently derived in [35], showing a major dependence with certain parameters such as the packet size in bits, the distance between the nodes, and the channel model parameters (mainly the path-loss exponent). In what follows, message exchange procedures and related communication cost expressions are presented for both architectures. Packet re-transmissions are not included in the cost models, which are based on physical-layer considerations.

#### 2.3.1. Centralized Architecture

**Figure 2:**
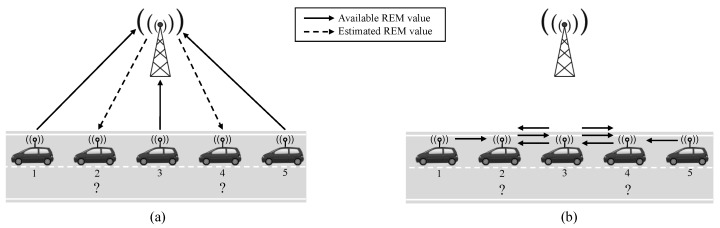
Example of the proposed reconstruction architectures in a platoon of five vehicles. (**a**) Centralized, (**b**) distributed. Vehicles 1, 3 and 5 access actual radio environment map (REM) values, while estimates are needed for vehicles 2 and 4.

Figure 2a,b show the message exchange procedure for centralized and distributed REM reconstruction via OK interpolation, respectively, in a platoon example with five vehicles. For simplicity, the platoon is in a symmetric position, but applying the same idea to the asymmetric case is straightforward. This example considers that the P=3 available REM values are at vehicles #1, #3 and #5, either through channel measurements or by accessing a previously stored REM of the area, while estimates are needed for vehicles #2 and #4. Note that the initial stage to acquire the REM values between the BS and vehicles #1, #3 and #5 using pilot transmissions is common to both architectures and hence, it has been intentionally omitted for simplicity in the figure.

In the centralized architecture in Figure 2a, the BS receives in a first step the P=3 REM values (represented by solid arrows) to perform the interpolation task. After the interpolation scheme is applied, the BS reports the N−P estimates to the vehicles of interest. In the example, the number of estimates is just 2 REM values (dashed arrows). As a result, the total number of exchanged messages equals *N* in a general case.

The communication cost for the centralized scheme, according to [35], can be calculated as:

(6) $${\mathrm{Cost}}_{\mathrm{cen}}=\sum_{i=1}^{N}\left(\frac{2^{B}-1}{d_{i}^{-\alpha }}\right),$$

where *B* is the packet size in bits, α is the path-loss exponent, and $d_{i}$ denotes the Euclidean distance between the BS and platoon vehicle #i, which can be calculated from the geometry in Figure 1 as follows:

(7) $$d_{i}=\frac{R+w/2}{\cos(\phi_{i})}.$$

#### 2.3.2. Distributed Architecture

In the distributed case (see Figure 2b), REM reconstruction is carried out directly at the vehicles with P=3 available REM values received by message passing from other vehicles in the group. In order to minimize the number of exchanged messages, each vehicle is assumed to obtain the closest available REM values. Theoretically, the range of transmission for each vehicle is limited, but the distance *D* is assumed to fulfill this value. Moreover, multi-hop communication is possible to communicate between non-adjacent platoon vehicles, if needed. In the example of Figure 2b, vehicle #2 would receive the REM values available at vehicles #1 and #3 from two direct messages, and the value available at vehicle #5 from a multi-hop transmission through vehicles #4 and #3. A similar approach is followed to provide the three REM values to vehicle #4.

It can be observed that all the messages are transmitted though one-hop communications between neighboring vehicles. As a result, messages traverse exactly the same distance, given by the separation between antennas of consecutive vehicles (*D*). Considering the unavailability of REM values in the first and last vehicle, which is the worst-case in terms of message exchange, the total number of exchanged messages in the distributed setup is P(N−1), and the estimated cost:

(8) $${\mathrm{Cost}}_{\mathrm{dis}}=P\left(N-1\right)\left(\frac{2^{B}-1}{D^{-\alpha }}\right).$$

In the case where the REM values of the first and last vehicle are available, the number of exchanged messages is reduced to P(N−3)+2.

## 3. Semivariogram Modeling

Once the REM values are available at the BS (in the centralized architecture), or at each vehicle (in the distributed case), the first step to reconstruct the REM in the remaining positions of the platoon is to build the EV in order to provide a semivariogram modeling via a parametric fitting. Reference [36] presents seven semivariogram models to be used when interpolating with Kriging, the spherical, the exponential and the Gaussian models being the most used in the literature of channel estimation. In scenarios with a random deployment of nodes with channel measurement capabilities, such as the ones considered in [10,11,19,22,37], different strategies are followed when selecting the semivariogram model. Some authors do not specify the model (see, e.g., [11]), whereas others assume the exponential model due to its correlation with the lognormal shadowing assumption (i.e., [10,19,24]). Reference [37] evaluates the Gaussian and spherical models, and the selection is performed through the comparison of the minimum square error between the estimated and the actual channel values. In [22], the fitting is performed via Matlab (www.mathworks.com) programming, obtaining that the power model is the most suited for their scenario. In this work, we deal with two particular features in the model selection step: (a) the number of available REM values to build the EV is small and limited to *P*, and (b) there is a certain fixed geometry in the EV calculations, which impacts on the model fitting, as it will be next discussed.

### 3.1. Semivariogram Models

In this contribution, we focus on the basic models as presented in [36]: spherical, exponential and Gaussian. The spherical model follows the expression:

(9) $$\bar{\gamma }\left(h\right)=\left\{\begin{array}{cc} c_{1}+c_{2}\left\{\frac{3}{2}\left(\frac{h}{c_{3}}\right)-\frac{1}{2}{\left(\frac{h}{c_{3}}\right)}^{3}\right\}, & 0\leq h<c_{3},\\ c_{1}+c_{2}, & c_{3}\leq h, \end{array}\right.$$

where $c_{1}$ and $c_{3}$ are nugget and range variables, respectively, while $c_{1}+c_{2}$ is known as sill. Theoretically, at a lag equal to zero, the semivariogram value should be zero. However, sometimes the semivariogram shows a nugget effect, with a value greater than zero, which is related to uncertainties and measurement errors. The range given by $c_{3}$ specifies the distance at which the model gets flat in the first place, and it indicates the spatial correlation limit. Finally, the sill is the value on the y-axis where the semivariogram attains the range. The exponential model is expressed as:

(10) $$\bar{\gamma }\left(h\right)=c_{1}+c_{2}\left\{1-\exp\left(-\frac{3h}{c_{3}}\right)\right\},\;0\leq h,$$

while the Gaussian is given by:

(11) $$\bar{\gamma }\left(h\right)=c_{1}+c_{2}\left\{1-\exp\left(-3{\left(\frac{h}{c_{3}}\right)}^{3}\right)\right\},\;0\leq h.$$

Once the model is selected, the parameters need to be determined by fitting the semivariogram model to the EV. Available methods include maximum likelihood estimation (MLE), weighted least squares estimation (WLSE) and generalized least squares estimation (GLSE) [36,38]. For the sake of lowering the complexity while retaining statistical efficiency, the WLSE is employed here. As recommended in [36], if there is no prior knowledge about variations in the EV, the following default values should be used as initialization:

(12) $$\begin{array}{c} c_{1_{ini}}=\max\left[0,\hat{\gamma }\left(h_{1}\right)-\frac{h_{1}}{h_{2}-h_{1}}\left(\hat{\gamma }\left(h_{2}\right)-\hat{\gamma }\left(h_{1}\right)\right)\right],\\ c_{3_{ini}}=\frac{h_{P}}{2},\\ c_{1_{ini}}+c_{2_{ini}}=\frac{\hat{\gamma }\left(h_{P-2}\right)+\hat{\gamma }\left(h_{P-1}\right)+\hat{\gamma }\left(h_{P}\right)}{3}, \end{array}$$

where $c_{1_{ini}}$, $c_{3_{ini}}$ and $c_{1_{ini}}+c_{2_{ini}}$ are the initial values for nugget, range and sill, respectively, while *P* is the number of available samples for the REM reconstruction.

### 3.2. Model Selection

We focus on a snapshot of the platoon service communication, where the vehicles are static in either a symmetric or asymmetric position (see Figure 1). The parameters of a 3GPP-like highway scenario are considered, where the BS is located R=35m away from the road border and each lane has a width w=4m [27]. The number of platoon vehicles *N* is set to 10. Two particular cases for the inter-vehicle distance are evaluated: L=2m (high-density platooning) and L=10m (normal platooning) [1]. A typical vehicle length of l=4.7 m is assumed. The original REM comprises the received power values at each of the 10 vehicle positions, obtained according to Equations (3) and (5) particularized as in [27]. More specifically, transmitted power is set to $P_{t}=10$ dBm, the constant path-gain factor is $K_{dB}=-137\;$dB for $d_{0}=1$ km, path-loss exponent takes the value α=3.7, and the shadowing contribution is modeled following a zero mean lognormal distribution with 8 dB standard deviation.

To evaluate the trade-off between signaling reduction when acquiring the REM versus quality of the estimation, we consider in Section 4 a number of available REM values to perform the reconstruction ranging from P=3 (minimum value to carry out OK as specified in Equation (12)) to P=9 (case where all the vehicles except one can access REM values). Note that *P* is directly related to the signaling reduction percentage. For instance, with N=10, reconstructing the REM with P=3 samples saves 70% of pilots.

The small size of the available set of data samples (3≤P≤9) is one of the factors impacting on the accuracy of the semivariogram model selection. Moreover, as depicted in Figure 1, when the platoon passes through the assisting BS, the set of distances between the platoon vehicles and the BS ranges between the ones given in the asymmetric case (maximum) and the ones obtained in the symmetric case (minimum). Besides, the vehicles in the latter case remain at the same distances with respect to the BS in both halves of the platoon (for instance, vehicles #1 and #N are at the same distances). Let us assume that the available REM values needed to calculate the EV from Equation (1) are due to the average received power, i.e., the path-loss contribution in our model. In this particular case, the field values are deterministic and directly related to the distance with respect to the BS. This simplified setting can be regarded as the worst case scenario in the semivariogram model step, since the EV in the symmetric case will not show an increasing trend with the lag distance (note that vehicles located at both ends of the platoon, which are the points at largest lag distance, will return exactly the same field value).

**Figure 3:**
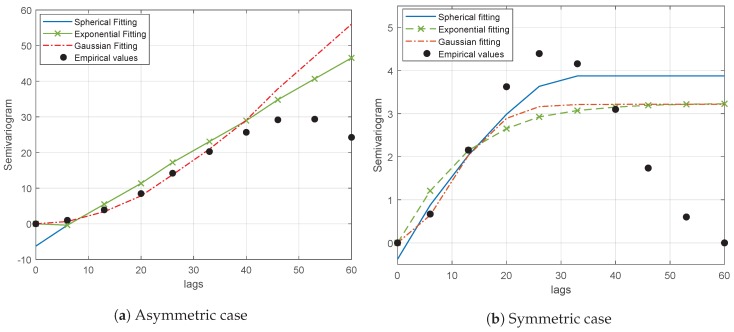
Example of spherical, exponential and Gaussian semivariogram modeling fitting versus empirical semivariogram.

Figure 3a,b represent examples of semivariogram fittings with the spherical, exponential and Gaussian models for the asymmetric and symmetric cases, respectively. As it can be seen, whereas the trend of the empirical values is reasonably well followed by the fitting models in the asymmetric case (Figure 3a), none of the models can obtain a good fitting in the symmetric case (Figure 3b), due to the particular geometry of the scenario under consideration. Although the random variation added by the shadowing contribution will in practice mitigate this effect, the average received power term is given by the path-loss contribution. Thus, for the sake of generality, the fitting is performed considering the average received power as the field value.

Two parameters are considered in order to determine which is the best semivariogram model to fit the available set of data: (a) the mean square error (MSE) between the EV ($\hat{\gamma }\left(h\right)$) and the corresponding values obtained after fitting with the models presented in Section 3.1 (${\bar{\gamma }}_{fit}\left(h\right)$); and (b) the Akaike information criterion (AIC) [36], which deals with the trade-off between the goodness of fit of the model and its simplicity [39]. The MSE is evaluated over the set comprising all lag distances as follows:

(13) $$\mathrm{MSE}=\sum_{N(h)}{\left(\hat{\gamma }\left(h\right)-{\bar{\gamma }}_{fit}\left(h\right)\right)}^{2},$$

while the AIC value is obtained by the following expression:

(14) $$\mathrm{AIC}=n\ln\left(\frac{\mathrm{MSE}}{n}\right)+2p,$$

where *n* is the number of points in the semivariogram (*P* in our case), ln(·) is the natural logarithm and *p* is the number of parameters in the models provided in Section 3 (p=3 corresponding to $c_{1}$, $c_{2}$ and $c_{3}$). The semivariogram fitting is performed in MATLAB.

Note that, for a given *P*, in this example there are 10P possible combinations or patterns of selected vehicles with available REM values. The results represent the average after testing all the possible patterns in each case. Table 1 shows the MSE and AIC for the three semivariogram models under study applied to the symmetric case. Note that, when fitting with more than one model, the one with the smallest AIC is the best model. It can be observed that, for both evaluated *L* values, the spherical model offers the best fitting for every *P*. The MSE and AIC results for the asymmetric case are presented in Table 2, showing that, in this setup, the Gaussian model outperforms the spherical and exponential ones. Therefore, following these results, the spherical model is chosen in the symmetric scenario, while the Gaussian model is the one being evaluated in the asymmetric case. REM reconstructed channel values will be obtained in a second step by applying the prediction given by Equation (2).

## 4. Performance Evaluation

In this section, patterns of vehicles’ positions with available REM values that provide the best REM reconstruction are first obtained for the scenario parameters and selected semivariogram models of Section 3.2. Second, the signaling reduction versus REM reconstruction accuracy of OK is assessed. Finally, communication cost results for the considered centralized and distributed architectures are presented.

### 4.1. Vehicle Selection

The REM reconstruction capability of OK in the symmetric and asymmetric scenarios was evaluated with the aim of establishing optimal patterns of vehicles’ positions for *P* values ranging between 3 and 9. For the sake of generality, the simulations were performed considering the averaged received power (i.e., path-loss contribution). As previously indicated, for a given number of available REM values to perform the interpolation (*P*) among N=10 vehicles, there were 10P possible combinations or patterns of selected vehicles. For each possible combination, the MSE between the actual field value and the estimated value was calculated, and the best combination was recorded, the latter understood as the one achieving the minimum MSE. Assuming that the set S defines the positions where the REM needs to be reconstructed for each *P* value and each possible combination, the MSE is given by:

(15) $$\mathrm{MSE}\left({\mathrm{dB}}^{2}\right)=\frac{1}{\left(N-P\right)}\sum_{x_{0}\in S}{\left(\hat{V}\left(x_{0}\right){|}_{P}-V\left(x_{0}\right)\right)}^{2}.$$

Recall that $x_{0}$ stands for the positions where the REM needs to be reconstructed and the cardinality of the set S is |S|=N−P.

**Figure 4:**
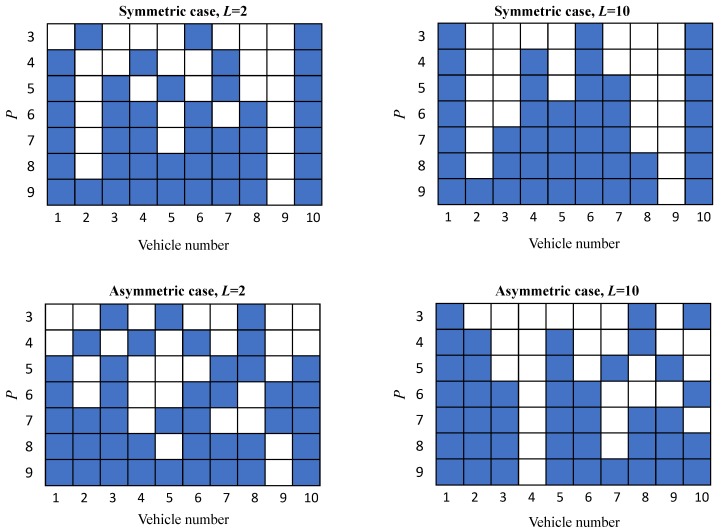
Optimal patterns of vehicles’ positions with available REM values that achieve the minimum MSE of REM reconstruction for different values of *P* in a platoon with N=10.

Figure 4 shows the patterns of vehicles’ positions with available REM values that provide the minimum MSE of REM reconstruction for different values of *P*. The selected vehicle positions are highlighted in blue. Recall that results for the OK in the symmetric scenario were obtained with the spherical model for the semivariogram, whereas the Gaussian model is used in the asymmetric case. It is worth noting that the minimum MSE was also achieved by considering the mirrored patterns. It can be observed that the selected vehicle pattern varied depending on the scenario (symmetric/asymmetric) and inter-vehicle distance value. Comparing first the results in the symmetric scenario, at the top of Figure 4, a clear trend to select the first and last vehicle samples together with an increasing number of additional positions generally located in the central area of the platoon was observed. Furthermore, for L=10 in this symmetric scenario, the selected combination for a given *P* always contained the same vehicles selected for P−1 plus an additional one. The latter effect was also observed for L=2, but starting from P=6. Regarding the vehicle patterns for the asymmetric case, at the bottom of the figure, the selected combinations were again included in the majority of cases the first and last vehicles, but did not follow a clear trend for a given *P* to include the same vehicles selected for P−1 plus an additional one.

### 4.2. REM Reconstruction Accuracy

In this evaluation, the field value was generated following the model from Equation (3), where lognormal shadow fading samples with zero mean and 8 dB standard deviation were added to the average received power values. We explored the effect of the shadowing correlation distance ($L_{corr}$) by choosing multiples of the separation between antenna positions in the platoon (*D*) for this parameter. In particular, $L_{corr}$ ranges from *D*, which generated totally uncorrelated shadowing samples between consecutive vehicle positions, to 7D, the latter representing that the shadowing samples of the first seven vehicles are correlated. In this way, for L=2, the maximum value $L_{corr}=7D$ led to a correlation distance of 46.9 m, approximating the value of 50 m considered in [27]. The shadowing correlation was generated through interpolation using a Matlab function based on splines. For instance, $L_{corr}=3D$ was modeled by setting independent shadow fading samples in positions 0,3D,6D and 9D, and obtaining the samples in the remaining locations through the spline interpolation.

Prior art has already justified the advantages of OK interpolation with respect to nearest neighbor, inverse distance weighting, natural neighbor and thin plate splines approximations (see the references discussed in Section 1). Hence, in the evaluation, we include the comparison with an alternative method based on cubic interpolation requiring also P≥3. In particular, the Matlab function based on the piecewise cubic Hermite interpolating polynomial (Pchip) is used. The modified Akima version of such interpolation (makima) was also tested, but since it performs worse than Pchip in our scenario, the comparison uses Pchip interpolation as a benchmark.

The accuracy of REM reconstruction was evaluated by calculating the MSE as per Equation (15). Both the minimum and average MSE values were evaluated by considering all the possible patterns of vehicles’ positions for each *P*. Each point in the figures is obtained by running 1000 simulations to average the effect of the shadowing.

**Figure 5:**
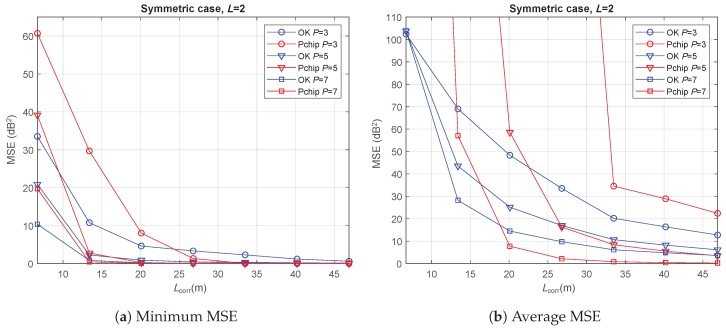
Path-loss and shadowing estimation results versus shadowing correlation distance in the symmetric case with L=2 m.

For the symmetric case with L=2, Figure 5a shows the minimum MSE values of REM reconstruction with OK and Pchip interpolation for P=3,5 and 7. Note that the first $L_{corr}$ point in the curves corresponds to the results with fully uncorrelated shadowing samples, meaning that in this case the methods cannot benefit from the shadow fading correlation to perform an adequate interpolation. According to P=3 curves, OK outperformed Pchip for $L_{corr}<4D$ (up to 27 m approx.). After this point, the Pchip wasis able to provide lower MSE values, the REM reconstruction MSE being very small for both techniques. In fact, for $L_{corr}>D$, the minimum MSE for the Kriging method was around 10 dB${}^{2}$ in the worst case (P=3), and below this value for the rest of configurations, which is consistent with previously reported results [22]. For P=5 and P=7, OK provided better results than Pchip when the shadowing was uncorrelated ($L_{corr}=D$), whereas both approaches presented similar results for the rest of shadowing correlation values.

Average MSE values for the same setup are shown in Figure 5b. It is worth noting that, at the point with totally uncorrelated channel samples, the MSE of OK was practically independent of the number of available REM values, due to the lack of correlation between samples.

When comparing OK and Pchip, it can be observed that, for all the *P* values, Pchip led to a very high MSE in a substantial number of $L_{corr}$ values, showing problems to carry out the interpolation for some vehicle combinations. On average, OK appeared to be more robust to selecting a suboptimum combination, providing MSE values upper bounded by 100. Moreover, it outperformed Pchip for N=3 in all cases, for N=5 when $L_{corr}\leq 4D$, and for N=7 when $L_{corr}\leq 3D$.

**Figure 6:**
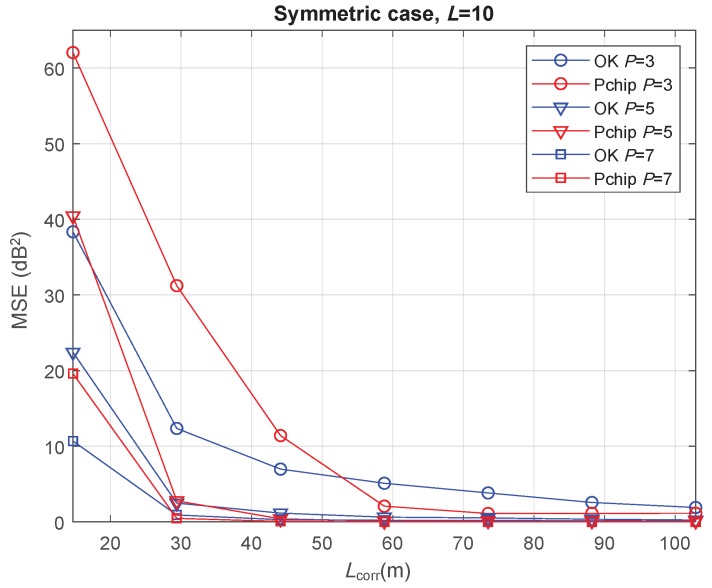
Minimum MSE of path-loss and shadowing estimation versus shadowing correlation distance in the symmetric case with L=10 m.

Figure 6 shows the minimum values for the MSE, considering the symmetric case after increasing the inter-vehicle distance to L=10. The range of $L_{corr}$ values now covers greater distances because of an increased value of *D*. Although the MSE was in some cases slightly higher than in the L=2 case, the conclusions remained the same. Thus, *L* is not a critical parameter for the REM reconstruction under the system model established in this paper.

**Figure 7:**
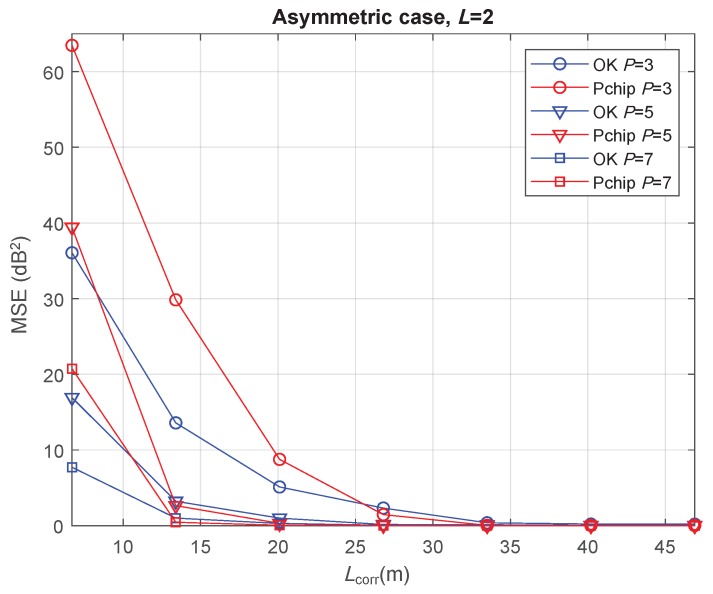
Minimum MSE of path-loss and shadowing estimation versus shadowing correlation distance in the asymmetric case with L=2 m.

Finally, the asymmetric case is evaluated with L=2. The resulting minimum MSE values are those shown in Figure 7. It is observed that, for a high correlation between the channel samples ($L_{corr}\geq 5D$), the REM reconstruction was highly accurate, since minimum MSE values went down to zero for every *P* value in both OK and Pchip. Note that, the MSE curves in the symmetric case for P=3 showed small values, remaining above zero in the whole $L_{corr}$ range (see Figure 5a and Figure 6). The slightly worse reconstruction accuracy in the symmetric scenario is then consistent with the semivariogram fitting discussion in Section 3, where the symmetric case experienced a poorer EV fitting due to the particular features presented in that section.

### 4.3. Communication Cost

The worst-case scenario to carry out the reconstruction is considered in order to narrow down the alternatives in the cost evaluation, that is, only P=3 REM values are available. The derived cost expressions in Equations (6) and (8) are evaluated for a number of platoon vehicles ranging from 4 to 10. As in the previous subsections, two values for the *L* parameter are considered (2 and 10). In these evaluations the parameter *B* is set to 8 bits, i.e., 1 byte, which allows to represent up to 256 different quantified power values.

**Figure 8:**
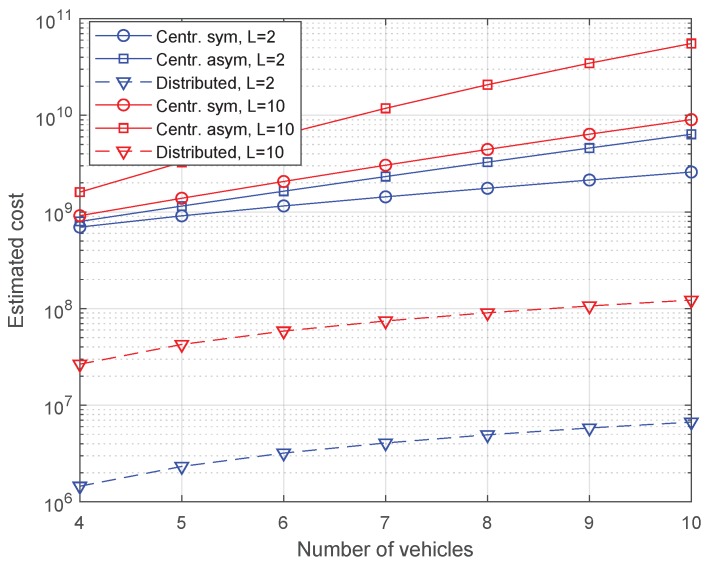
Estimated cost versus number of vehicles for the centralized (symmetric and asymmetric) and distributed REM reconstruction schemes with L=2 m and L=10 m.

Figure 8 shows the estimated cost versus number of platoon vehicles for the centralized and distributed approaches, the former one considering both a symmetric and an asymmetric scenario. The results indicate that both centralized setups showed a linear increase, whereas the distributed scheme increased in a logarithmic fashion. Regarding the comparison between the symmetric and asymmetric cases in the centralized architecture, the communication cost values were, for a given number of vehicles, higher in the asymmetric setup. Besides, the cost gap between symmetric and asymmetric cases increased linearly with the number of platoon vehicles. This trend is justified by the fact that, for a given value of *N*, the distance contributions by vehicles from 1 to N/2 in the asymmetric setup are approximately the same as those by vehicles from N/2+1 to *N* in the symmetric setup, but the remaining half of vehicles in the asymmetric case contribute with double distances with respect to the symmetric case.

Focusing now on the effect of the inter-vehicle distance (*L*), the results show that it had a higher impact on the distributed scheme, showing a constant cost gap of more than an order of magnitude for all numbers of vehicles. This result is reasonable, as the distance used for the cost estimation is straightforwardly related to this parameter. It can be further observed that the communication costs of the distributed approach were substantially lower than those calculated for both centralized schemes, despite the fact that the distributed approach exchanged a larger number of messages (e.g., 23 messages for N=10). This is due to the higher proximity between the communication points, which increases the denominator term in the cost functions. Recall that *L* does not contribute to the cost of the centralized schemes directly, but combined with a term that includes also the distance from the BS to the road border *R* (see Equation (7)). Therefore, the contribution of *L* turns more significant as its magnitude approaches the one of *R*. The latter effect also justifies that the cost gap between centralized and distributed schemes decreases as *L* gets higher for a given *N*.

It has to be noted that the costs in Figure 8, which are related to the message exchange in the REM reconstruction phase, contributed only in part to the total costs for channel estimation. In practice, these costs need to be added to the costs of initially sending pilots from the BS to the vehicles. In the worst-case, the BS sent a different pilot to every platoon vehicle (*N* messages), while, in the proposed approach, both in the centralized and distributed architectures, the BS sent only P<N pilots (saving N−P messages). Equation (8) directly provides the cost of transmitting one message from the BS to each member of the platoon (*N* messages). This equation can also straightforwardly provide the cost of transmitting the pilot messages to only a subset of *P* vehicles.

Let us assume the values N=10, P=3 and the best evaluated case in terms of communication cost, that is, L=2. First, considering the asymmetric setup, the cost to initially send *N* pilots in the conventional scheme is $6.4\cdot 10^{9}$, whereas the cost to send *P* pilots in the proposed approach was $2.3\cdot 10^{9}$, leading to a cost reduction of 64%. Second, in the symmetric setup with the same particular values, the costs to initially send *N* and *P* pilots were $2.6\cdot 10^{9}$ and $1\cdot 10^{9}$, respectively, involving a cost reduction of 62%. Furthermore, the absolute cost reductions in both setups (in the order of $10^{9}$) compensated the cost for reconstructing the REM in the proposed distributed scheme (in the order of $10^{7}$, see Figure 8), supporting the proposed REM reconstruction scheme. As a result, we can conclude that the REM reconstruction scheme can effectively reduce the signaling for channel estimation while keeping the MSE below 10 dB${}^{2}$ for the values under consideration.

## 5. Conclusions

This work proposes the use of radio environment map (REM) reconstruction techniques to reduce the control information dedicated to the channel acquisition stage in platoon-based cellular vehicle to anything (V2X) communications. Assuming that a minimum number of REM values are available at a subset of the platoon vehicles, Ordinary Kriging (OK) spatial interpolation is then applied to reconstruct the values in the remaining ones. In a first step, different semivariogram fitting functions are evaluated. It is observed that the semivariogram fitting is sensitive to the relative positions between the BS and the platoon vehicles, which requires to consider two different evaluation scenarios separately, here called as symmetric and asymmetric. The fitting results conclude that a spherical model is more suitable in the symmetric scenario, whereas a Gaussian model provides the best fitting in the asymmetric case. This conclusion reinforces the need to carefully perform the semivariogram modeling step when OK is applied as a spatial interpolation technique, and it even paves the way for a dynamic and adaptive semivariogram modeling.

After selecting the proper semivariogram fitting, results on the trade-off between REM reconstruction accuracy and signaling reduction are provided. It is shown that the accuracy is highly affected by the selected vehicle positions with available REM values, leading to different optimum vehicle positions’ patterns for the symmetric and asymmetric scenarios. The general trend for these optimum patterns, as the number of available REM values increases, is to include the values for the first and last platoon vehicles, together with an increasing number of available samples in the central area.

The OK technique has been later compared to interpolation based on cubic Hermite polynomials (Pchip) in terms of reconstruction accuracy, showing OK superior results at low to medium shadowing correlation distances with the minimum number of available samples. At high correlation distances, both techniques achieve very similar reconstruction results with negligible MSE. The OK technique is also more robust to selecting suboptimum vehicle patterns, as justified by its bounded average MSE values.

Finally, a communication cost evaluation related to REM reconstruction in centralized and distributed architectures is provided, showing that the centralized scheme in the asymmetric scenario involves the highest costs. Considering also the cost of initial pilot transmissions from the BS in the analysis, it is observed that, for N=10, P=3 and L=2, the proposed approach can effectively reduce the signaling for channel estimation while keeping the MSE of REM values below 10 dB${}^{2}$.

The work presented in this exploratory paper paves the way towards enabling advanced physical-layer functionalities for the platoon V2X scenario. More precisely, two particular use cases are envisaged by the authors of this paper: mobile relays (where the entire platoon would cooperate with the base station to improve the communication link of a poor coverage vehicle outside the platoon); and intra-platoon relaying, where platoon vehicles with good quality channels would act as relays for platoon vehicles with low quality channels. To this end, our future work will address the need to include time effects via stochastic modeling of the platoon location, as well as exploring the capability of advanced forms of Kriging such as Regression Kriging. A more complete analysis of the signaling reduction versus performance trade-off will be also carried out through system-level evaluations.

## Figures and Tables

**Table 1:** Semivariogram modeling (mean square error (MSE) and Akaike information criterion (AIC)) in the symmetric case for L=2 and L=10. Minimums are highlighted in violet color.

	Spherical		Exponential		Gaussian	
L=2	**MSE**	**AIC**	**MSE**	**AIC**	**MSE**	**AIC**
P=3	2.59	5.56	4.15	6.97	4.09	6.93
P=4	2.43	4.01	4.22	6.21	3.93	5.93
P=5	2.08	1.61	3.64	4.41	3.31	3.94
P=6	1.47	−2.44	2.94	1.72	2.59	0.96
P=7	0.90	−8.36	2.23	−2.00	1.95	−2.95
P=8	0.54	−15.57	1.76	−6.11	1.43	−7.77
P=9	0.31	−24.32	1.46	−10.37	1.14	−12.60
L=10	**MSE**	**AIC**	**MSE**	**AIC**	**MSE**	**AIC**
P=3	122.49	17.13	248.56	19.25	226.98	18.98
P=4	127.76	19.86	260.69	22.71	230.58	22.22
P=5	115.53	21.70	229.26	25.13	205.41	24.58
P=6	87.75	22.10	194.89	26.88	174.08	26.21
P=7	60.08	21.05	158.15	27.82	138.80	26.91
P=8	42.53	19.37	133.84	28.54	111.24	27.06
P=9	32.35	17.51	118.10	29.17	94.52	27.16

**Table 2:** Semivariogram modeling (MSE and AIC) in the asymmetric case for L=2 and L=10. Minimums are highlighted in violet color.

	Spherical		Exponential		Gaussian	
L=2	**MSE**	**AIC**	**MSE**	**AIC**	**MSE**	**AIC**
P=3	18.07	11.37	14.82	10.79	4.98	7.52
P=4	20.00	12.44	17.79	11.97	5.08	6.96
P=5	19.83	12.89	18.06	12.42	4.92	5.92
P=6	19.10	12.95	17.69	12.49	3.90	3.42
P=7	18.10	12.65	16.67	12.07	2.64	−0.83
P=8	17.29	12.17	15.79	11.44	1.58	−6.98
P=9	16.18	11.28	15.71	11.01	0.80	−15.78
L=10	**MSE**	**AIC**	**MSE**	**AIC**	**MSE**	**AIC**
P=3	278.89	19.60	228.51	19.00	41.83	13.91
P=4	292.43	23.17	261.93	22.73	35.65	14.75
P=5	296.11	26.41	269.92	25.94	31.15	15.15
P=6	293.34	29.34	272.68	28.90	22.70	13.98
P=7	291.02	32.10	275.34	31.70	14.57	11.13
P=8	285.06	34.59	268.62	34.11	9.38	7.27
P=9	284.00	37.10	269.47	36.59	6.37	2.89

## References

[1] Study on Enhancement of 3GPP Support for 5G V2X Services. https://itectec.com/archive/3gpp-specification-tr-22-886/.

[2] Nardini G., Virdis A., Campolo C., Stea A.M.G. (2018). Cellular-V2X Communications for Platooning: Design and Evaluation. Sensors.

[3] Roger S., Martín-Sacristán D., Garcia-Roger D., Monserrat J.F., Spapis P., Kousaridas A., Ayaz S., Kaloxylos A. (2019). Low-Latency Layer-2-Based Multicast Scheme for Localized V2X Communications. IEEE Trans. Intell. Transp. Syst..

[4] Martín-Sacristán D., Roger S., Garcia-Roger D., Monserrat J.F., Kousaridas A., Spapis P., Ayaz S., Zhou C. Evaluation of LTE-Advanced Connectivity Options for the Provisioning of V2X Services. Proceedings of the IEEE Wireless Communications and Networking Conference (WCNC).

[5] Marsch P., Bulakci O., Queseth O., Boldi M. (2018). 5G System Design: Architectural and Functional Considerations and Long Term Research.

[6] Amoozadeh M., Deng H., Chuah C.N., Zhang H.M., Ghosal D. (2015). Platoon management with cooperative adaptive cruise control enabled by VANET. Veh. Commun..

[7] Martín-Sacristán D., Roger S., Garcia-Roger D., Monserrat J.F., Kousaridas A., Spapis P., Zhou C. Signaling Reduction in 5G eV2X Communications Based on Vehicle Grouping. Proceedings of the 2019 European Conference on Networks and Communications (EuCNC).

[8] Tang X., Xu X., Svensson T., Tao X. (2017). Coverage Performance of Joint Transmission for Moving Relay Enabled Cellular Networks in Dense Urban Scenarios. IEEE Access.

[9] Fodor G., Roger S., Rajatheva N., Slimane S.B., Svensson T., Popovski P., Da Silva J.M.B., Ali S. (2016). An Overview of Device-to-Device Communications Technology Components in METIS. IEEE Access.

[10] Chowdappa V., Botella C., Samper-Zapater J.J., Martinez R.J. (2018). Distributed Radio Map Reconstruction for 5G Automotive. IEEE Intell. Transp. Syst. Mag..

[11] Yilmaz H.B., Tugcu T., Alagöz F., Bayhan S. (2013). Radio Environment Map as Enabler for Practical Cognitive Radio Networks. IEEE Commun. Mag..

[12] Kryszkiewicz P., Kliks A., Kułacz L., Bogucka H., Koudouridis G.P., Dryjański M. (2018). Context-Based Spectrum Sharing in 5G Wireless Networks Based on Radio Environment Maps. Wirel. Commun. Mob. Comput..

[13] Perez-Romero J., Zalonis A., Boukhatem L., Kliks A., Koutlia K., Dimitriou N., Kurda R. (2015). On the Use of Radio Environment Maps for Interference Management in Heterogeneous Networks. IEEE Commun. Mag..

[14] Braham H., Jemaa S.B., Fort G., Moulines E., Sayrac B. (2016). Spatial Prediction Under Location Uncertainty in Cellular Networks. IEEE Trans. Wirel. Commun..

[15] Barman N., Valentin S., Martini M.G. Predicting Link Quality of Wireless Channel of Vehicular Users Using Street and Coverage Maps. Proceedings of the IEEE 27th International Symposium on Personal, Indoor and Mobile Radio Communications (PIMRC).

[16] Sybis M., Kryszkiewicz P., Sroka P. (2018). On the Context-Aware, Dynamic Spectrum Access for Robust Intraplatoon Communications. Mob. Inf. Syst..

[17] Dwarakanath R.C., Naranjo J.D., Ravanshid A. Modeling of Interference Maps for Licensed Shared Access in LTE-advanced Networks Supporting Carrier Aggregation. Proceedings of the 2013 IFIP Wireless Days (WD).

[18] Yilmaz H., Tugcu T. (2015). Location Estimation-based Radio Environment Map Construction in Fading Channels. Wirel. Commun. Mob. Comput..

[19] Üreten S., Yongaçoğlu A., Petriu E. A Comparison of Interference Cartography Generation Techniques in Cognitive Radio Networks. Proceedings of the 2012 IEEE International Conference on Communications (ICC).

[20] Taranto R.D., Muppirisetty S., Raulefs R., Slock D., Svensson T., Wymeersch H. (2014). Location-aware Communications for 5G Networks: How Location Information Can Improve Scalability, Latency, and Robustness of 5G. IEEE Signal Process. Mag..

[21] Chowdappa V.P., Fröhle M., Wymeersch H., Botella C. Distributed Channel Prediction for Multi-agent Systems. Proceedings of the 2017 IEEE International Conference on Communications (ICC).

[22] Han Z., Liao J., Qi Q., Sun H., Wang J. (2019). Radio Environment Map Construction by kriging Algorithm Based on Mobile Crowd Sensing. Wirel. Commun. Mob. Comput..

[23] Dall’Anese E., Kim S.J., Giannakis G. (2011). Channel Gain Map Tracking via Distributed Kriging. IEEE Trans. Veh. Technol..

[24] Sato K., Fujii T. (2017). Kriging-Based Interference Power Constraint: Integrated Design of the Radio Environment Map and Transmission Power. IEEE Trans. Cogn. Commun. Netw..

[25] Babak O., Deutsch C. (2009). Statistical Approach to Inverse Distance Interpolation. Stoch. Environ. Res. Risk Assess..

[26] Chowdappa V., Botella C., Beferull-Lozano B. Distributed Clustering Algorithm for Spatial Field Reconstruction in Wireless Sensor Networks. Proceedings of the 2015 IEEE 81st Vehicular Technology Conference (VTC Spring).

[27] Study on LTE-Based V2X Services. https://www.tech-invite.com/3m36/tinv-3gpp-36-885.html.

[28] Schabenberger O., Gotway C.A. (2004). Statistical Methods for Spatial Data Analysis.

[29] Goldsmith A. (2005). Wireless Communications.

[30] Gudmunson M. (1991). Correlation Model for Shadow Fading in Mobile Radio Systems. Electron. Lett..

[31] Katagiri K., Sato K., Fujii T. (2018). Crowdsourcing-Assisted Radio Environment Database for V2V Communication. Sensors.

[32] Huang Y., Hua Y. (2009). Energy Planning for Progressive Estimation in Multihop Sensor Networks. IEEE Trans. Signal Process..

[33] Shah S., Beferull-Lozano B. (2013). Joint Sensor Selection and Multihop Routing for Distributed Estimation in Ad-hoc Wireless Sensor Networks. IEEE Trans. Signal Process..

[34] Frantzis F., Chowdappa V., Botella C., Samper J.J., Martinez R.J. Radio Environment Map Estimation Based on Communication Cost Modeling for Heterogeneous Networks. Proceedings of the 2017 IEEE 85th Vehicular Technology Conference (VTC Spring).

[35] Roger S., Botella C., Meza-Sánchez E.E., Pérez-Solano J.J. Communication Cost of Channel Estimation Interpolation for Group-based Vehicular Communications in Cellular Networks. Proceedings of the 10th Euro American Conference on Telematics and Information Systems (EATIS).

[36] Jian X., Olea R., Yu Y.S. (1996). Semivariogram Modeling by Weighted Least Squares. Comput. Geosci..

[37] Boccolini G., Hernández-Peñaloza G., Beferull-Lozano B. Wireless Sensor Network for Spectrum Cartography Based on Kriging Interpolation. Proceedings of the IEEE 23rd International Symposium on Personal, Indoor and Mobile Radio Communications (PIMRC).

[38] Cressie N.A.C. (1993). Statistics for Spatial Data.

[39] Akaike H. (1974). A New Look at the Statistical Model Identification. IEEE Trans. Autom. Control.

